# Membrane-bound estrogen receptor alpha initiated signaling is dynamin dependent in breast cancer cells

**DOI:** 10.1186/s40001-018-0328-7

**Published:** 2018-06-07

**Authors:** Istvan Marczell, Petra Balogh, Gabor Nyiro, Anna L. Kiss, Balazs Kovacs, Gabor Bekesi, Karoly Racz, Attila Patocs

**Affiliations:** 1 2nd Department of Medicine, Semmelweis University, Budapest, Szentkirályi utca 46., 1088 Hungary; 2 Department of Human Morphology and Developmental Biology, Semmelweis University, Budapest, Hungary; 3 Molecular Medicine Research Group, Hungarian Academy of Sciences, Budapest, Szentkirályi str. 46., 1088 Hungary; 4 Department of Aquaculture, Szent Istvan University, Godollo, Hungary; 5 HAS-SE ‘Lendület’ Hereditary Endocrine Tumors Research Group, Hungarian Academy of Sciences, Semmelweis University, Budapest, 46. Szentkiralyi str, 1088 Hungary; 6 Department of Laboratory Medicine, Semmelweis University, Budapest, Nagyvárad sq 4, 1089 Hungary

**Keywords:** MCF-7, Estrogen–BSA, Estrogen membrane receptor, mER

## Abstract

**Background:**

Although membrane-associated estrogen receptors (mERs) have been known to play important role in steroid-induced signal transmission, we still know little about their function in the estrogen-induced proliferation of breast cancer cells.

**Methods:**

In our current work we tried to separate membrane-initiated estrogen receptor signaling from the overall estrogenic effect in MCF-7 breast carcinoma cells. Re-analyzing expression data from multiple microarray experiments, we selected a set of key regulatory genes involved in proliferation regulation and estrogen signaling to monitor estrogen-induced transcription changes. We then compared these expression changes after 17β-estradiol and a membrane receptor selective estrogen–BSA treatment using quantitative real-time PCR. In order to follow receptor trafficking we used light and electron microscopy.

**Results:**

Our quantitative real-time PCR results confirmed that the selective membrane receptor agonist, estrogen–BSA induces similarly pronounced expression changes regarding these genes as 17β-estradiol. Morphological study revealed that the membrane-bound form of classical estrogen receptor alpha is internalized after ligand binding via dynamin-dependent, caveola-mediated endocytosis. Inhibition of this internalization with dynamin inhibitor, dynasore practically abolished the regulatory effect of E2-BSA, suggesting that interaction and internalization with the scaffold protein is necessary for effective signaling.

**Conclusions:**

The physiological role of plasma membrane estrogen receptor alpha is intensively studied, yet there are still several aspects of it to be resolved. The dynamin-dependent, ligand-mediated internalization of mERs seems to play an important role in estrogen signaling. Our results may serve as another example of how membrane initiated estrogen signaling and nuclear receptor initiated signaling overlap and form an intertwined system.

**Electronic supplementary material:**

The online version of this article (10.1186/s40001-018-0328-7) contains supplementary material, which is available to authorized users.

## Background

The genomic effects of estradiol are traditionally mediated by nuclear estrogen receptors (ERs). According to the classical view, after ligand binding, ERs (ER-α, ER-β) are translocated to the nucleus where they bind to the DNA cooperating with a number of co-regulators that together influence gene expression patterns [1, 2]. However, in the last two decades it became apparent that a subpopulation (10–15%) of ERs are membrane-bound and this receptor pool also contributes to the overall estrogenic effect. A subset of these receptors are palmitoylated membrane-bound forms (or splice variants) of the classical nuclear receptors [3], while there is a structurally different receptor type, called GPER (G-protein-coupled estrogen receptor, formerly known as GPR30) [4]. Upon ligand binding, these structurally diverse membrane receptors induce a variety of rapid changes in cellular functions via second messenger pathways through which they also contribute to the transcriptional effects of estrogen, including the regulation of proliferation [5], cell migration, and development [6]. These effects are mostly transmitted via the activation of alternative MAP kinase pathways [7–10].

Compared to nuclear receptors, membrane-bound ERs show different signaling kinetics and are more exposed to various stimuli such as paracrine, autocrine, or endocrine signals. These differences render membrane estrogen receptors ideal pharmacological targets in cancer therapy.

The plasma membrane ER-alpha (ERα) pool has been described to be predominantly localized in a certain type of lipid rafts called caveolae, where the receptor interacts with the scaffold protein caveolin-1 [11]. Caveolae are 50–100 nm flask or omega-shaped plasma membrane invaginations that have crucial role in endocytosis, transcytosis, and provide a platform at the cell surface for various signaling events [12–15]. The two main structural proteins of caveolae are caveolin-1 and caveolin-2 proteins [16, 17]. Caveolin-1 is known to be involved in trafficking of the estrogen receptor alpha to and from the cell surface and in maintaining an environment where coupling between signaling partners is possible [18].

In our current work we aimed to dissect the nuclear- and membrane-initiated estrogenic effect in MCF-7 cells using molecular and cell biological methods. By re-analyzing publicly available gene expression data from various databases, we primarily selected a set of key regulatory and signaling molecules involved in E2-related proliferation regulation of MCF-7 cells. The expression profile of key signaling (ERBB2, KDM4B) and regulatory genes (MYC, CCND1, KCNK5) were compared after treatment with E2 or membrane selective estrogen-like compounds with and without the concomitant inhibition of membrane receptor internalization [19]. We not only confirmed that membrane-impermeable estrogen–BSA has a significant effect on gene expression which is comparable to E2, but also demonstrated that this effect is linked to dynamin-dependent receptor endocytosis. The morphological results further proved that dynamin inhibition corrupts receptor internalization and prevents further signaling as well.

## Methods

### Cell culturing

Human breast carcinoma MCF-7 cell line was obtained from the cell bank of the 2nd Department of Pathology, Semmelweis University. Cells were grown in RPMI-1640 medium (Sigma-Aldrich, Cat. No.: R8758-500ML) supplemented with 10% fetal bovine serum, 100 U/mL penicillin, and 100 μg/mL streptomycin in a humidified incubator at 37 °C with 5% CO_2_. Prior to treatments, cells were cultured in serum and antibiotic-free medium for 24 h.

### Treatments

E2-BSA (Sigma-Aldrich, V St. Louis, MO, USA) was dissolved in phosphate-buffered saline (PBS), and free estradiol was removed by filtration using the technique described by Stevis et al. [20]. The filtered solution was added to serum and antibiotic-free medium. Treatments were carried out in three different concentrations (10^−10^ M, 10^−9^ M, 10^−8^ M), and each group consisted of three samples. Estrogen concentrations were calculated with 30 mol steroid per mol bovine serum albumin (BSA) according to manufacturer’s specifications.

17β-Estradiol (Sigma-Aldrich, St. Louis, MO, USA) was dissolved in ethanol then added to serum and antibiotic-free medium in the same three concentrations (10^−10^ M, 10^−9^ M, 10^−8^ M). G1 (Tocris Bioscience, Bristol, UK) was dissolved in dimethyl sulfoxide (DMSO) in the final concentration of 10^−8^ M. Dynasore (Sigma-Aldrich) was dissolved in DMSO and used in 80 µmol treatment concentration, and was applied 30 min prior to subsequent 17β-estradiol treatment (in 10^−10^ M concentration). All treatments were performed in triplicate.

The reagents were added to the cell media for 3 h with the same dissolvent, and then cells were collected and kept at − 80 °C in Trizol^®^ Reagent (Applied Biosystems, Life Technologies, Carlsbad, California, US) until further processing.

### Gene expression studies

#### Re-analysis of gene expression studies available in microarray data repositories

Microarray data were downloaded from Gene Expression Omnibus (GEO; http://www.ncbi.nlm.nih.gov/geo/). Four time-course studies with a total of 18 arrays were selected for further analysis (see Additional file 1: Table Sheet 1). The treatment period was 3 or 4 h for all samples. Two common gene chip families (Affymetrix U133 and U133 Plus 2.0) were used in these experiments. There are 22,277 common probe sets between the two array types that map 13,186 genes. For the meta-analysis, the common probe sets across the platforms were used. All data were normalized with the Guanine Cytosine Robust Multi-Array Analysis (GCRMA) method [21].

The statistical significance of the results was evaluated by the non-parametric algorithm ‘Rank products,’ available as the ‘RankProd’ package at Bioconductor (http://www.bioconductor.org). This statistically robust method has been demonstrated as a reliable tool for microarray data analysis [22]. It detects genes that are consistently highly ranked in several replicated experiments, independently of their numerical intensities. The method ranks each feature within an experiment based on that features’ score (in our case Log expression values), and then combines these ranks, instead of combining the data or *p* values. The results are provided in the form of *p* values defined as the probability that a given gene is ranked in the observed position by chance. Differentially regulated probe sets were selected based on the estimated percentage of false-positive predictions (pfp), which is equivalent to a false discovery rate [23]. The pfp is calculated using a permutation-based procedure (50,000 permutations were conducted). Genes with a pfp of less than 0.05 were selected for further investigation (Additional file 1: Table Sheet 2).

On GEO and ArrayExpress we found one microarray experiment consisting of 4 arrays carried out with estrogen–BSA-treated MCF-7 cells. Measurements were conducted with Affymetrix U133 Plus 2 platform, and normalized with GeneChip Robust Multiarray Averaging (GCRMA) method. Statistical analysis was carried out using Student’s *t* test, and *p* < 0.05 was considered significant (Results can be found in Additional file 1: Table Sheet 3).

#### Gene expression changes upon E2, E2-BSA, G1, and dynasore treatments

Total RNA was isolated from an average of 2 × 10^6^ MCF-7 cells cultured in T-25 flasks. RNA purification was carried out using Trizol^®^ Plus RNA Purification Kit (Life Technologies, Carlsbad, California, USA) according to the manufacturer’s instructions. RNA content was determined using NanoDrop 2000 (Thermo Scientific, Waltham, MA, USA) spectrophotometer. For each sample ~ 1000 ng RNA was transcribed to cDNA using High Capacity RNA-to-cDNA Kit (Applied Biosystems, Foster City, CA, USA). Pre-designed TaqMan^®^ Gene Expression Assays (Applied Biosystems) were used for real-time PCR (CCND1—Hs00765553_m1; ERBB2—Hs01001580_m1; GAPDH—Hs99999905_m1; CKNK5—Hs00186652_m1; KDM4B—Hs00943636_m1; MYC—Hs00153408_m1; RPL13a—Hs04194366_g1). We used GAPDH and RPL13A as housekeeping genes [24]. Measurements were performed using TaqMan^®^ Fast Advanced Master Mix with no UNG. The 7500 Fast Real-Time PCR System (Life Technologies, Carlsbad, California, USA) was used with the following parameters: 95 °C for 20 s followed by 60 two-step cycles at 95 °C for 3 s and at 60 °C for 30 s. All RT-PCRs were performed in triplicate. SDS 1.3.1 software (Applied Biosystems, Foster City, CA, USA) was used for calculation of the threshold cycle (Ct) values in each sample. For interpretation of the results, we applied the ddCT method [25]. The results were adjusted to RPL13A as a selected housekeeping gene [24]. Statistical analysis for single dose treatments (in our study treatments with G1 and co-treatment using dynasore with 17β-estradiol) was carried out using Student’s *t* test. Samples showed equal variance, and significance was considered at *p* < 0.05.

### Imaging studies

#### Preparation of semithin and ultrathin cryosections

For morphological examination control and treated MCF-7 cells were fixed in freshly prepared 4% paraformaldehyde (PFA) in 0.1 M PB for 1 h at room temperature. The PFA-fixed samples were placed and stored in 1% paraformaldehyde (in 0.1 M PB) at 4 °C until further processing. For cryosectioning and immunolabeling, the fixed cells were subsequently detached with a scraper, washed twice in PBS and once in 0.02 M glycine/PBS by centrifugations at 1000 rpm for 10 min each at room temperature. The pellets were then infiltrated with 12% gelatine in PB at 37 °C for 10 min and then centrifuged with 1000 rpm at room temperature for 5 min. The samples were placed on ice for 30 min and afterwards cut into small blocks. For cryoprotection, the blocks were infiltrated with 2.3 M sucrose at 4 °C overnight and afterwards mounted on metal pins, frozen, and stored in liquid nitrogen. For preparing semithin and ultrathin cryosections we used Leica Ultracut S ultramicrotome equipped with cryo-attachment (Vienna, Austria). The pickup solution was a 1:1 mixture of 2.3 M sucrose and 1.8% methylcellulose.

#### Immunolabeling for light and electron microscopy

The 0.7 μm semithin cryosections mounted on microscopic slides were incubated with 0.02 M glycine in PBS for 15 min and were blocked in PBS containing 1% BSA. Primary antibodies rabbit polyclonal anti-caveolin-1 antibody (1:200; BD, Transduction Laboratories, Lexington, KY) and rabbit polyclonal anti-ERα (H-184): sc-7207) antibody (1:200, Santa Cruz Biotechnology, Inc) were applied in 1% BSA-containing buffer in a humidified chamber at 4 °C (overnight). Biotinylated anti-rabbit IgG (1:200; Vector Laboratories Inc, Burlington, CA) was applied as a secondary antibody for indirect immunolabeling when two polyclonal primary antibodies were used. For immunofluorescence visualization, Streptavidin Alexa Fluor conjugated to 488 (1:200) was used and for double immunolabeling the second primary antibody was visualized with goat anti-rabbit IgG Alexa Fluor 555 (1:200, Molecular Probes, Leiden, the Netherlands). The nuclei were stained with DAPI (Vector Laboratories Inc, Burlington, CA). The visualization was performed in a Bio-Rad (Zeiss, Budaörs, Hungary) Radiance 2100 Rainbow Confocal Scanning system coupled to a Nikon Eclipse E800 microscope (Nikon, Tokyo, Japan). Lasersharp 2000 6.0 software (Zeiss, Oberkochen, Germany) was used for image acquisition and final images were assessed using Adobe Photoshop 7.0. program (San Diego, CA, USA). (Only linear adjustments with respect to brightness and contrast were applied to the entire image that did not alter the interpretation of the original material.) Before double immunolabeling all antibodies were rigorously tested for single labeling at different dilutions. Negative controls were applied in each experiment to avoid false-positive results. For cryosectioning and immuno-EM, the fixed tissues were further processed as described by Slot and Geuze [23] and the detailed description of the labeling process can be found in Ref. [15].

## Results

### Re-analysis of gene expression studies available at microarray data repositories

Out of the 22,277 probe sets, we identified 378 at 3 or 4 h time point, with a very strict false discovery rate of pfp ≤ 0.05 (see “Methods”). By mapping the probe sets to genes, we have identified 285 unique upregulated and 49 downregulated genes that were differentially expressed. (All results obtained from microarray meta-analysis are available in Additional file 1: Table Sheet 1.) The selected key genes (KCNK5, KDM4B, MYC, and CCND1) were all significantly upregulated while ERBB2 (HER2) was downregulated, although its expression change was not significant compared to untreated cells. We further focused on the expression of the above-mentioned five genes as their expression has already been extensively studied and all have been confirmed as a target of E2.

Statistical analysis of the microarray data of estrogen–BSA treatment identified 586 genes with significantly altered expression. MYC, KDM4B, and KCNK5 were significantly upregulated after 3 h of estrogen–BSA treatment. The expression change of CCND1 did not reach statistical significance similarly to the downregulated ERBB2.

### Validation of gene expression changes upon E2, E2-BSA, G1, and dynasore treatments by qRT-PCR

**Fig. 1:**
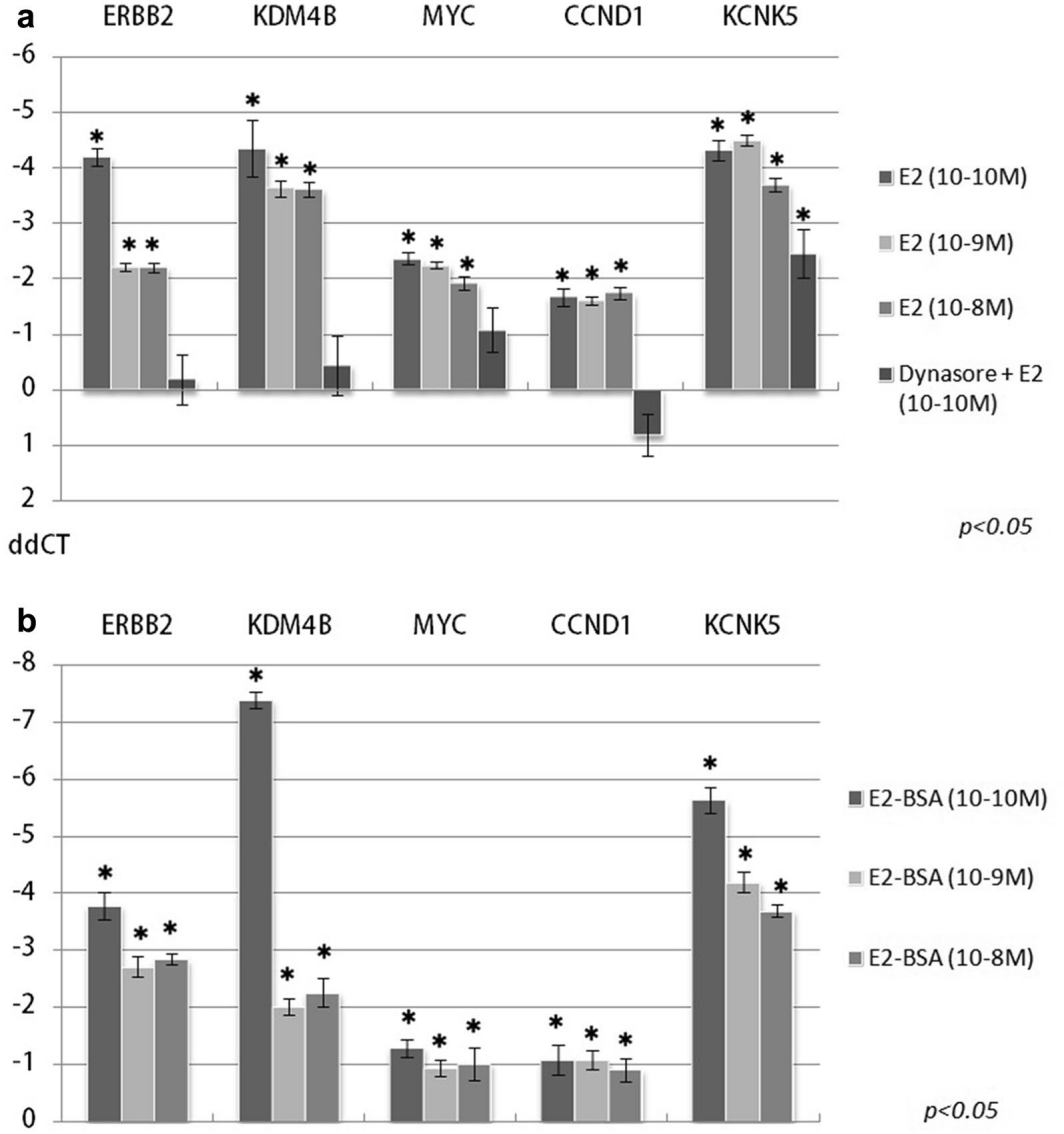
Gene expression changes after treatment of MCF-7 cells with 17β-estradiol and estrogen–BSA. Expression changes of CCND1, ERBB2, KCNK5, KDM4B, and MYC after 17-β-estradiol (E2) (**a**) and estrogen–BSA treatment (**b**). Treatments were performed in three different concentrations (10^−10^ M, 10^−9^ M, 10^−8^ M) and a similar E2 treatment (10^−10^ M) on dynamin inhibitor (dynasore, 30 min) pretreated cells. *Y*-axis represents ddCT values, 0 line indicates control level. (Error bars represent standard deviation, asterisks indicate significant changes compared to control with *p* value < 0.05). Numerical ddCT values are shown in Additional file 1: Table Sheet 5

Upon 17β-estradiol treatment, the expression of all genes selected from microarray studies was significantly upregulated (Fig. 1a). Membrane receptor selective estrogen–BSA (mER) treatment also upregulated remarkably the expression level of all these genes and this change was similar or even stronger for KCNK5 and KDM4B compared to those observed with 17β-estradiol treatment (Fig. 1b). Pretreatment with the dynasore abolished the effect of 17β-estradiol on all genes except of KCNK5, although its expression was also significantly lower than measured after 17β-estradiol treatment (Fig. 1a).

**Fig. 2:**
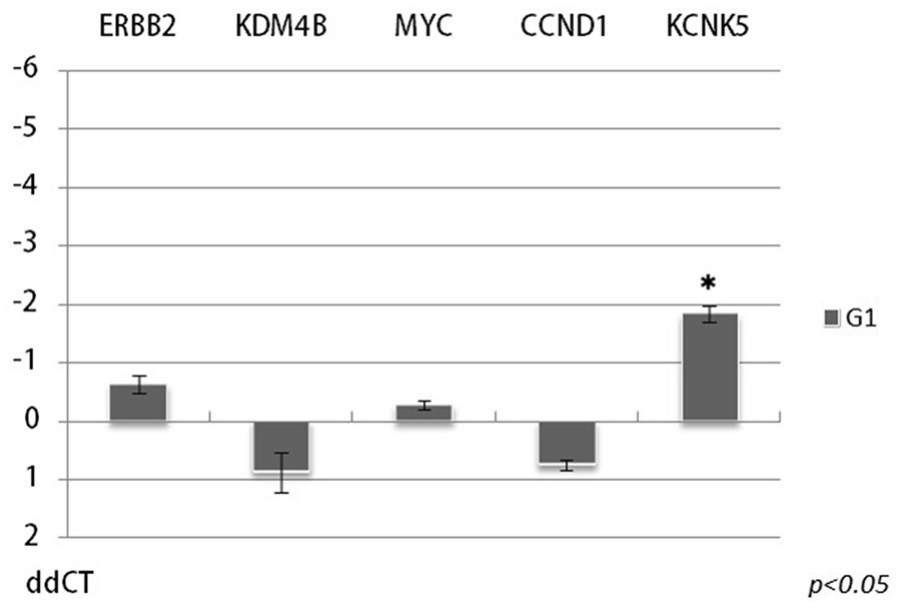
Expression changes of CCND1, ERBB2, KCNK5, KDM4B, and MYC after selective GPER agonist, G1 treatment. A significantly lower expression changes were observed compared to estrogen or estrogen–BSA treatments. *Y*-axis represents ddCT values, 0 line indicates control level. (Error bars represent standard deviation, asterisks indicate significant changes compared to control with a *p* value < 0.05). Numerical ddCT values are shown in Additional file 1: Table Sheet 5

In order to dissect the mERα signaling from GPER signaling, we treated MCF-7 cells with G1, a selective GPER agonist. G1 treatment of MCF-7 cells resulted only moderate effect on expression of the studied genes. Significant effect was observed for KCNK5 only (Fig. 2). Numerical ddCT values are shown in Additional file 1: Table Sheet 5.

### Imaging studies

**Fig. 3:**
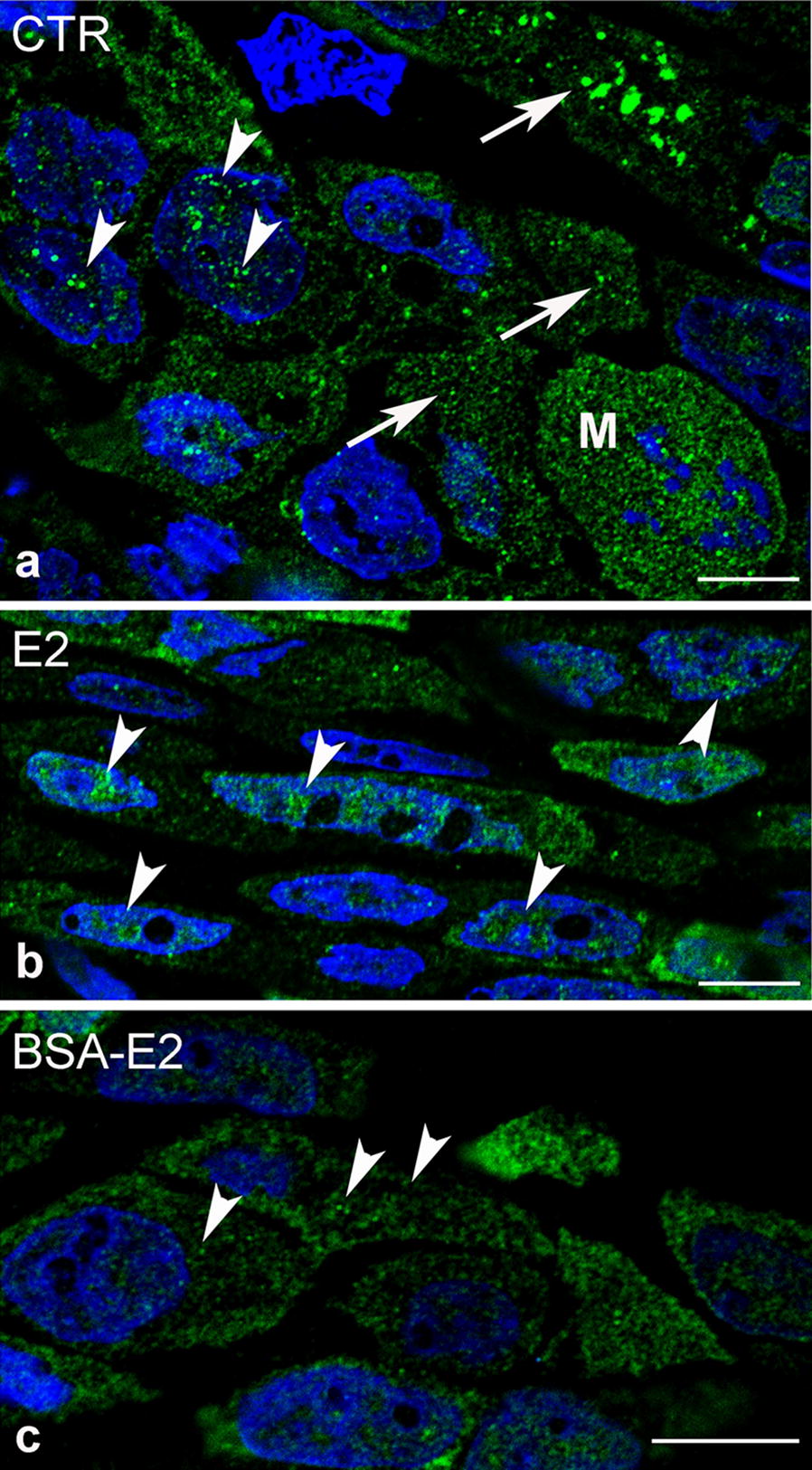
ER-α labeling of control and treated MCF-7 cells on semithin frozen sections. **a** ER-α receptor labeling occurred in aggregates or as punctate structures both inside the nucleus (arrowheads) as well as in the cytoplasm of untreated MCF-7 cells. Observe a mitotic form (M) where intensive ERα expression could be detected. **b** Upon E2 treatment (2 min, 10^−8^ M/L), the majority of receptor labeling accumulated inside the nucleus (arrowheads). **c** Immunofluorescence labeling of ER-α could predominantly be observed in the cytoplasm and submembranous localization of MCF-7 cells upon BSA-E2 treatment (arrowheads). Nuclei were stained with DAPI, bars indicate 10 µm

We examined ERα distribution in control and treated MCF-7 cells using fluorescence immunolabeling. We detected ERα signals both inside the nucleus as well as in the cytoplasm of MCF-7 cells under control conditions (Fig. 3a). Upon E2 treatment the majority of receptor labeling accumulated inside the nucleus, while after estrogen–BSA stimulation ERα could be observed predominantly in the cytoplasm and to a lesser extent in the plasma membrane (Fig. 3b, c).

**Fig. 4:**
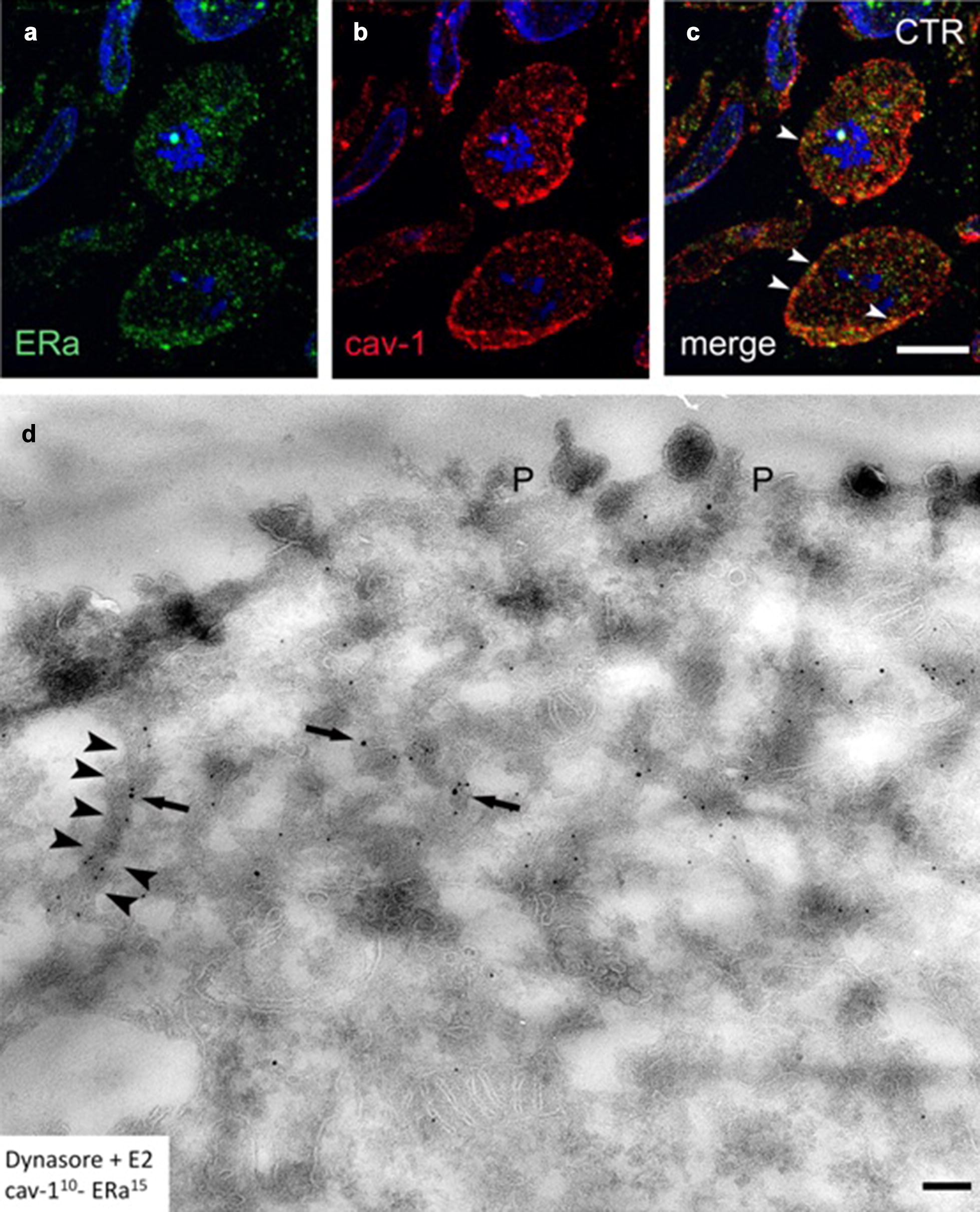
Morphological changes upon combined Dynasore-E2 treatment of MCF-7 cells. Semithin frozen sections of control MCF-7 cells labeled with antibodies directed against ER-α (green) and caveolin-1 (red). **a**–**c** ER-α receptor labeling occurred inside the cytoplasm and the plasma membrane in MCF-7 cells. Nuclear occurrence of the receptor could also be observed, however, to a lesser extent. The merged image of ER-α and caveolin-1 double labeling shows overlapping areas at the plasma membrane (arrowheads), while intracytoplasmic co-labeling could also be detected. Bars indicate 10 µm, nuclei were stained with DAPI. **d** Ultrathin cryosection shows morphologically distorted, elongated caveolae (arrowheads) indicating the effect of Dynasore treatment. Larger gold particles labeling ER-α could be observed in the close vicinity of caveolin-1 positive structures right beneath the plasma membrane (P). Note that the deeper cytoplasmic areas lack both small (ERα) and larger (caveolin-1) gold particles indicating the disturbed internalization of caveolin-1 positive vesicles. Bar indicates 200 nm

In order to prove that plasma membrane ERα pool resides in caveolin-1 positive lipid rafts, we carried out double immunolabeling. Our result showed several orange puncta indicating co-labeling of caveolin-1 and ERα. This could be observed both along the plasma membrane as well as in the cytoplasm (Fig. 4a–c).

Further, we examined the morphological effect of combined dynasore and estrogen treatment of MCF-7 cells with immunoelectron microscopy. After dynasore treatment, ERα labeling occurred in distorted, elongated caveolin-1 positive structures right beneath the cytoplasm. The deeper cytoplasmic areas lacked caveolin-1 positive vesicular structures suggesting that endocytosis via this route was disturbed (Fig. 4d).

## Discussion

Three subtypes (ERα, ERβ, and GPER) and several isoforms of estrogen receptors have been detected in various cells and tissues that mediate genomic and non-genomic estradiol effects that influence both nuclear and cytoplasmic events. Based on the localization of the receptor and the availability of ligands, nuclear (mostly genomic) and extra-nuclear (mostly non-genomic) actions have been described. Both pathways have a role in proliferation regulation of breast cancer cells, hence anti-estrogen therapies have been successfully used in the management of estrogen receptor-positive breast cancers [26].

Nuclear receptor initiated signaling mainly affects genes containing estrogen responsive elements (ERE) in their promoters. However, a significant number of genes without ERE have also been reported to be affected by estrogen treatments suggesting a non-classical action of estrogens. In the last few decades it became apparent that many of these expression changes can be attributed to membrane-bound estrogen receptors that can be activated with membrane-impermeable estrogenic compounds [27].

In our current study we aimed to clarify the effect of the membrane-bound ER on gene expression of MCF-7 cells. We completed a complex gene expression study to identify key signaling molecules in the estrogen-induced proliferative response. We collected and reanalyzed whole genome expression data publicly available in various gene expression databases, and individually validated the selected genes after treatments with estrogen and two membrane selective estrogen agonists. In a parallel morphological study we followed estrogen receptor alpha trafficking with and without dynamin inhibition using light and electron microscopy.

Meta-analysis of microarray experiments confirmed the importance of membrane-initiated estrogen signaling in MCF-7 cells, as the membrane selective estrogen–BSA treatment led to similar expression changes as estrogen—20% of the genes altered by 17β-estradiol was also significantly altered by E2-BSA (the list of the overlapping genes are shown in Additional file 1: Table Sheet 4) [28–30]. These data also showed that the expression changes of these genes occurred in a relatively short time period.

Individual measurement of genes by qRT-PCR experiments confirmed and validated the microarray data. Treatment of MCF-7 cells with E2-BSA led to similar expression changes observed after 17β-estradiol treatment regarding KDM4B, MYC, KCNK5, and CCND1 but not ERBB2.

KDM4B is a master regulator in the estrogen-induced signaling cascade and its depletion attenuates breast cancer development in vitro and in vivo [31]. Its upregulation by membrane-associated ER could mean a novel therapeutic or diagnostic opportunity and calls for further investigation. Myc [32], KCNK5 [33], and CCND1 [34] are also important mediators of the estrogen-induced signaling cascade. All these molecules have been identified as central hubs in the estrogen signaling network [23]. Of all these genes KCNK5 looks particularly interesting because its expression was upregulated not only by E2 and E2-BSA but also after G1 treatment. It is accepted that KCNK5 not only has a functionally active ERE in its promoter region but it is under the regulation of estrogen in MCF-7 cells. It is less clear though, if the potassium channel coded by this gene plays a pathophysiological role [33] or the upregulation is the result of coregulation with other estrogen-induced genes [35]. Our data suggest that the transcription of KCNK5 is under regulation of both the classical nuclear receptor and the membrane-bound receptor pool including GPER. Although several rapid effects of E2 have been attributed to GPER [4], in our model this was the only gene to be significantly upregulated by G1. It appears that in this setting at least the role of GPER may be inferior or different from classical ER-induced signaling.

Our quantitative RT-PCR results did not confirm the upregulation of ERBB2 predicted by the results of the microarray meta-analysis. We believe that this is the consequence of the gene’s complex regulation. Previously Hurtado et al. demonstrated that ERBB2 is under the regulation of estrogen signaling, but in a rather complex way, orchestrated by a number of co-factors and transcription factors [36–38].

Imaging results demonstrated that mER undergoes ligand-mediated receptor internalization via a dynamin-dependent route. Dynasore being the inhibitor of dynamin, blocks the scission of membrane vesicles and interferes with lipid raft organization and occasionally membrane receptor signaling [39, 40]. Dynamin inhibition with dynasore prior to 17β-estradiol stimulation resulted in submembranous accumulation of ERα in distorted and elongated caveolae. Quantitative RT-PCR results showed that dynasore pretreatment drastically decreased estrogen-induced transcriptional changes confirming that signaling from the membrane estrogen receptor complex was disturbed.

The plasma membrane and cytoplasmic co-localization of ERα with caveolin-1 that we observed in our study confirms the previous results showing that mER-alpha pool was situated in caveolin-1 positive lipid rafts and in pinched off caveolae [27]. Our results obtained by imaging studies are in line with those obtained by Zivadinovic et al. [29] showing that ER-alpha labeling occurs at the surface of non-permeabilized cells having high mER expression. They also demonstrated by Western analysis that in MCF-7 cells caveolin-1, caveolin-2, and ER-alpha colocalized in the same membrane sub.

According to our results estrogen-induced proliferative signaling involves both nuclear and membrane estrogen receptors. Among membrane estrogen receptors, ER-alpha and GPER, the former seems to be responsible for influencing primarily events triggered from the plasma membrane. We also managed to demonstrate that these membrane receptors reside in caveolae and that dynamin-mediated receptor internalization is an important step in membrane-initiated ER-alpha signaling.

Parallel regulation of such pivotal genes both from nuclear and membrane receptor structures suggests some kind of cooperation between these two receptor pools. Wheeling et al. [41] studying aldosterone rapid effects introduced the so-called ‘two-step model’ in which receptor structures mediating rapid aldosterone effects somehow modulate the genomic effects of the hormone. Regulation of key signaling genes from estrogen membrane receptors may also serve as a similar system and the examined ligand-mediated receptor internalization is a further proof how the two pathways could interact. An interesting result of our qRT-PCR experiment was that overstimulation of the membrane ER pool led to reverse dose dependency in gene expressions parallel to the receptor internalization. Downregulation of membrane-initiated signaling by ligand-mediated receptor internalization is a well-known regulatory mechanism that has been described in a variety of signaling pathways [42]. In that sense our results support the ‘two-step model’ in which estrogen signaling initiated from the surface and from the nuclear receptor pool together compose a regulatory system that helps in the adaptation of nuclear signaling to hormonal stimuli and protects it from overstimulation [43].

## Conclusions

In a broader context our work emphasizes that understanding the cooperation between different estrogen receptor-mediated pathways may bring us closer to understand the complex and integrated system by which the effects of estrogen signaling are completed. Thus, more effective pharmacological treatment options could be developed in the future for various diseases including breast cancer.

## Additional file

**Additional file 1.** Gene expression and microarray data.

## Abbreviations

BSA: bovine serum albumin
CCND1: cyclin D1
DMSO: dimethyl sulfoxide
E2: estradiol
ER: estrogen receptor
ERBB2: Erb-B2 Receptor Tyrosine Kinase 2
ERE: responsive elements
GCRMA: Guanine Cytosine Robust Multi-Array Analysis
GPER: G-protein-coupled estrogen receptor
KCNK5: potassium two pore domain channel subfamily K member 5
KDM4B: lysine demethylase 4B
MCF-7: Michigan Cancer Foundation-7, a human breast adenocarcinoma cell line
mER: membrane-associated estrogen receptor
MYC: Myc proto-oncogene
PBS: phosphate-buffered saline
PFA: paraformaldehyde
qRT-PCR: quantitative real-time polymerase chain reaction
